# Fluoride‐Oxalate and Lithium Heparin Anticoagulant Tubes Are Reliable Alternatives to EDTA for HbA1c Measurement

**DOI:** 10.1155/bmri/7743935

**Published:** 2026-08-20

**Authors:** Lamia Abdellaoui, Asma Krir, Manel Soltani, Aouatef Trabelsi, Mehdi Mrad, Afef Bahlous

**Affiliations:** ^1^ Department of Clinical Biochemistry, Pasteur Institute of Tunis, University of Tunis El Manar, Tunis, Tunisia, pasteur.tn

**Keywords:** anticoagulants, ethylenediaminetetraacetic acid, glycated hemoglobin, lithium heparin, preanalytical phase, sodium fluoride

## Abstract

**Background:**

The dual role of HbA1c in diagnosing and monitoring diabetes mellitus has driven a continuous increase in routine testing requests. To optimize laboratory workflows and avoid dedicated EDTA blood draws, this study is aimed at determining whether fluoride‐oxalate and lithium heparin anticoagulant tubes could serve as reliable alternatives to EDTA tubes for HbA1c measurement on the COBAS INTEGRA 400 plus analyzer.

**Methods:**

Over 2 months at the Pasteur Institute of Tunis, paired blood samples from patients undergoing routine HbA1c testing were analyzed. Standard K3‐EDTA tubes were compared with either lithium heparin or fluoride‐oxalate tubes using a turbidimetric inhibition immunoassay on a COBAS INTEGRA 400 plus analyzer. The study relied exclusively on residual clinical material without imposing specific preanalytical restrictions.

**Results:**

The study population comprised 277 patients, divided into Group 1 (K3‐EDTA vs. fluoride‐oxalate, *n* = 200) and Group 2 (K3‐EDTA vs. lithium heparin, *n* = 77). HbA1c results from both alternative tubes showed strong correlation with K3‐EDTA (*r* = 0.99, *p* < 0.0001), with no significant overall differences. Passing–Bablok regression revealed no constant or proportional bias for lithium heparin, whereas a systematic bias was observed for fluoride‐oxalate. Bland–Altman analysis of log‐transformed data indicated no significant mean bias for either fluoride‐oxalate (−0.15%; 95% LoA [−2.76% to +2.53%]) or lithium heparin (−0.04%; 95% LoA [−2.77% to +2.76%]), although a slight proportional bias was detected in both. Finally, HbA1c measurements in both tube types exhibited high diagnostic performance at clinical cutoffs (*p* < 0.0001).

**Conclusions:**

Fluoride‐oxalate and lithium heparin anticoagulant tubes provide reliable alternatives to standard K3‐EDTA tubes for HbA1c measurement, with no clinically meaningful differences observed. However, we emphasize that these results are strictly validated for the Roche COBAS immunoassay.

## 1. Introduction

Glycated hemoglobin type A1c (HbA1c) measurement is the gold standard for monitoring diabetes mellitus and is increasingly used as a diagnostic tool (1), leading to a continuous rise in routine testing requests. Conventionally, testing is performed on ethylenediaminetetraacetic acid (EDTA)–anticoagulated whole blood (2), historically preferred for its ability to preserve cellular integrity (3).

To optimize resource management and avoid additional blood draws, the use of alternative anticoagulants is highly desirable (4). Previous studies have investigated the feasibility of using lithium heparin or fluoride‐oxalate tubes for HbA1c measurement (5, 6). While some reports have demonstrated acceptable agreement, others have identified statistically significant biases, the clinical relevance of which remains variable. Preanalytical variables such as tube components, anticoagulants, and gels remain recognized sources of analytical variability (7).

Despite these previous efforts, data regarding the analytical agreement of these tubes specifically for the turbidimetric inhibition immunoassay (TINIA) method remain limited and require validation. Therefore, this study was designed to add new data by assessing the specific preanalytical interference of two commonly used anticoagulants in biochemistry laboratories.

Our objective was to determine whether fluoride‐oxalate and lithium heparin tubes could serve as reliable alternatives to EDTA tubes for HbA1c measurement on the COBAS INTEGRA 400 plus analyzer.

## 2. Materials and Methods

### 2.1. Subjects

Blood samples were collected over a 2‐month period at the phlebotomy center of the Pasteur Institute of Tunis. The study included patients undergoing routine HbA1c testing either for diabetes monitoring or screening for impaired glucose metabolism alongside other biochemical parameters. This approach ensured that the collected samples spanned the entire analytical measurement range.

For each patient, two 5 mL plastic Vacutest tubes (Vacutest Kima S.r.l., Italy) were vacuum‐drawn via peripheral venous phlebotomy: a standard tripotassium‐EDTA (K3‐EDTA) tube and a second tube containing either lithium heparin or sodium fluoride/potassium oxalate (fluoride‐oxalate), depending on the additional parameters requested. To avoid unnecessary blood draws, the study relied exclusively on residual clinical material; no supplementary tubes were collected specifically for comparative purposes.

Following collection, samples were immediately mixed by slow inversions and transported at ambient temperature to the biochemistry laboratory within 2 h, where they were remixed prior to analysis according to the manufacturer′s instructions. Samples were collected without specific preanalytical restrictions to preserve local‐routine clinical variability.

### 2.2. Ethical Clearance

This study was conducted within a continuous preanalytical quality improvement framework, in compliance with ISO 15189 requirements for pre‐examination processes (Clause 7.2) (4), and reported according to the Biospecimen Reporting for Improved Study Quality (BRISQ) guidelines (8). Consistent with the International Federation of Clinical Chemistry and Laboratory Medicine (IFCC) recommendations, the secondary use of anonymized leftover specimens for quality control and tube comparison was considered ethically acceptable, as no additional phlebotomy or blood collection was performed (9). This approach also aligns with the principles of the World Medical Association (WMA) Declaration of Taipei by ensuring zero additional risk to patients (10).

### 2.3. Methods

In this in vitro comparative study, whole blood HbA1c was measured using the Tina‐quant Hemoglobin A1c Gen.3 reagent on a COBAS INTEGRA 400 plus analyzer (Roche Diagnostics). This assay relies on a TINIA method (11) which is standardized according to the IFCC reference method and certified by the National Glycohemoglobin Standardization Program (NGSP), ensuring traceability to the Diabetes Control and Complications Trial (DCCT) and United Kingdom Prospective Diabetes Study (UKPDS) reference targets (12). For clinical relevance, results were reported in standard percentages (%) derived from the analyzer′s integrated IFCC‐to‐NGSP master equation.

To validate the analytical reliability for detecting minor differences between paired samples, assay precision was evaluated using PreciControl HbA1c (Roche Diagnostics). Within‐run precision (repeatability) demonstrated coefficients of variation (CV) of 1.0% at a mean HbA1c of 5.6% (Level 1) and 0.9% at a mean of 10.9% (Level 2). Additionally, to account for daily routine variability, between‐run precision (reproducibility) was assessed, yielding CVs of 1.2% and 1.0% for the same respective levels.

### 2.4. Statistical Analysis

Data normality was evaluated using the Shapiro–Wilk test. Given the nonnormal distribution, results were expressed as medians and interquartile ranges (IQRs). Correlations between K3‐EDTA and the alternative anticoagulant tubes were assessed using Spearman′s rank correlation, while differences between paired measurements were analyzed using the Wilcoxon signed‐rank test. Method comparability was investigated via Passing–Bablok regression and Bland–Altman analysis; the latter was performed on log‐transformed data due to the nonnormal distribution of residuals. Diagnostic performance (sensitivity and specificity) at clinical decision thresholds was evaluated using receiver operating characteristic (ROC) curve analysis (13–15). Statistical analyses were performed using IBM SPSS Statistics (Version 25.0) and MedCalc software. Statistical significance was set at *p* < 0.05.

## 3. Results

The study population comprised 277 patients with a median age of 59 years (IQR [48–68]) and a sex ratio of 0.82. The obtained HbA1c results covered nearly the entire measurement range, from 4.59% to 15.08%. Patients were divided into two comparative groups (Table 1): Group 1, patients evaluated using K3‐EDTA versus fluoride‐oxalate tubes; and Group 2, patients evaluated using K3‐EDTA versus lithium heparin tubes.

**Table 1:** Results of HbA1c measurements in the two study groups.

Group	Number of patients	Types of sampling tubes	Results of Hb1Ac	*p* value
1	200	K_3_‐EDTA	6.18 [5.55; 7.44]	*p* < 0.0001
		Fluoride‐oxalate	6.17 [5.56; 7.52]	
2	77	K_3_‐EDTA	5.84 [5.53; 7.29]	*p* < 0.0001
		Lithium heparin	5.88 [5.55; 7.40]	

For both groups, HbA1c results showed a strong correlation with those measured in K3‐EDTA tubes (*r* = 0.99, *p* < 0.0001). The median difference was 0, and no statistically significant difference was observed between the result distributions (*p* = 0.44 and *p* = 0.16, respectively).

**Figure 1:**
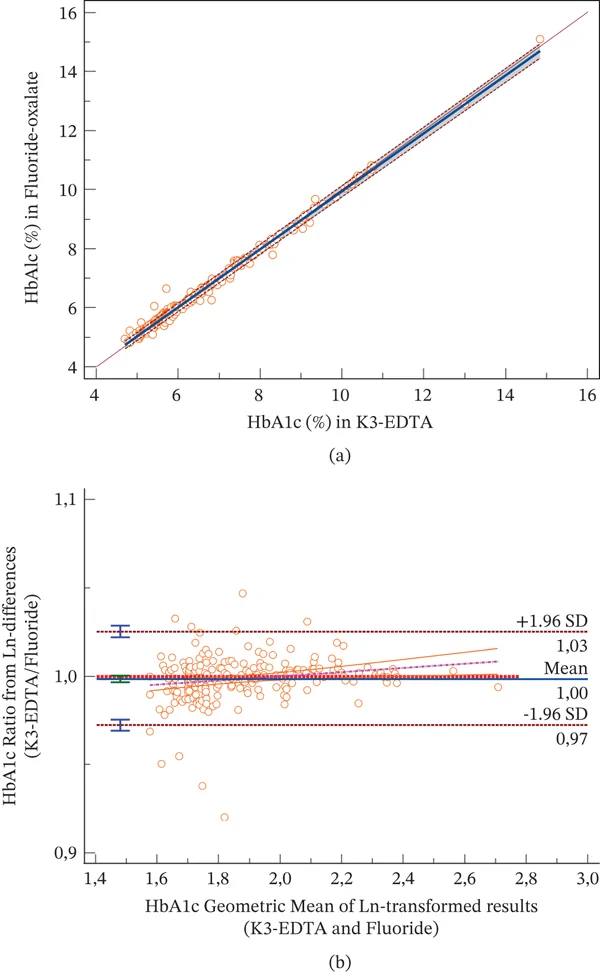
Comparison of HbA1c results in Group 1 (200 paired samples). (a) Passing–Bablok regression analysis comparing HbA1c measurements between K3‐EDTA and fluoride‐oxalate Vacutest tubes. The regression equation is *y* = 0.13 (95*%*CI [0.05–0.21]) + 0.98*x* (95% CI [0.96–0.99]). The solid line represents the regression, while the dashed lines indicate the 95% confidence interval. Comparison with the line of identity (dotted line, *x* = *y*) shows a constant bias (intercept 95% CI [0.05–0.21]) and a proportional bias (slope 95% CI [0.96–0.99]). (b) Bland–Altman plot comparing HbA1c measurements between K3‐EDTA and fluoride‐oxalate Vacutest tubes following logarithmic transformation. The mean bias (solid blue line) shows a ratio of 0.9985 [95% CI: 0.9966–1.0004], indicating no significant systematic bias (*p* = 0.12) relative to the line of equality (red line, ratio = 1). The limits of agreement (outer dotted brown lines) demonstrate that 95% of the differences fall within a narrow range of [−2.76%; +2.53%], confirming analytical concordance. However, regression analysis (pink dashed line) reveals a proportional bias (slope = 0.0119; *p* = 0.0062).

**Figure 2:**
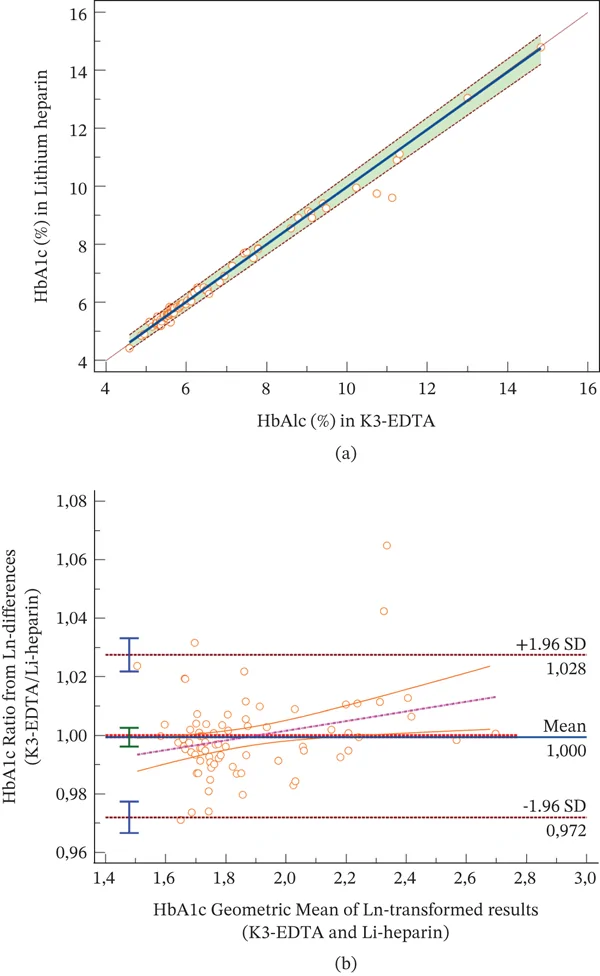
Comparison of HbA1c results in Group 2 (77 paired samples). (a) Passing–Bablok regression analysis comparing HbA1c measurements between K3‐EDTA and lithium heparin Vacutest tubes. The regression equation is *y* = 0.09 (95*%*CI [−0.03 to 0.25]) + 0.98*x* (95% CI [0.96–1.00]). The solid line represents the regression, while the dashed lines indicate the 95% confidence interval. Comparison with the line of identity (dotted line, *x* = *y*) shows no constant bias (intercept 95% CI [−0.03 to 0.25]) and no proportional bias (slope 95% CI [0.96–1.00]). (b) Bland‐Altman plot comparing HbA1c measurements between K3‐EDTA and lithium heparin Vacutest tubes following logarithmic transformation. The mean bias (solid blue line) shows a ratio of 0.9996 [95% CI: 0.9964–1.0028], indicating no significant systematic bias (*p* = 0.79) relative to the line of equality (red line, ratio = 1). The limits of agreement (outer dotted brown lines) demonstrate that 95% of the differences fall within a narrow range of [−2.77%; +2.76%], confirming analytical concordance. However, regression analysis (pink dashed line) reveals a proportional bias (slope = 0.0166; *p* = 0.0116).

Passing–Bablok regression analysis showed no constant or proportional bias for HbA1c results measured in lithium heparin compared to those measured in K3‐EDTA. However, based on the intercept and slope, a systematic bias (comprising both constant and proportional biases) was observed in HbA1c results measured in fluoride‐oxalate compared to those measured in K3‐EDTA (Figures 1A and 2A). No significant deviation from linearity was observed (*p* = 0.27 and *p* = 0.35, respectively).

Bland–Altman analysis indicated no significant mean bias. A mean bias of −0.15% (*p* = 0.12, 95% limits of agreement [LoA] [−2.76%; +2.53%]) was observed for HbA1c results measured in fluoride‐oxalate tubes and −0.04% (*p* = 0.78, 95% LoA [−2.77%; +2.76%]) for those measured in lithium heparin tubes. Since the confidence interval around the mean difference included zero, the mean bias was not statistically significant in either case. However, regression analysis of the differences revealed a statistically significant proportional bias for both fluoride‐oxalate (*p* = 0.006) and lithium heparin (*p* = 0.01) (Figures 1B and 2B).

Regarding individual differences, the Bland–Altman plots demonstrated a few outliers beyond the 95% LoA. For HbA1c measured in fluoride‐oxalate, 10 outliers were noted, with extreme deviations ranging from −7.9% to +4.3%. For the lithium heparin tubes, only four outliers were detected, ranging from −2.8% to +6.4%.

Evaluation of diagnostic performance revealed high accuracy for HbA1c measurements in fluoride‐oxalate and lithium heparin tubes at established decision thresholds (*p* < 0.0001) (Table 2).

**Table 2:** Analysis of diagnostic performance of HbA1c measurement on the different tubes.

Decision threshold		AUC	Sensitivity	Specificity
Diabetes: HbA1c ≥ 6.5*%* (13)	Group 1	0.99	95.4%	97.3%
		(95% CI [0.97–1.00], *p* < 0.0001)	(95% CI [88.8–98.7])	(95% CI [92.4–99.4])
	Group 2	0.99	84.6%	96.0%
		(95% CI [0.93–1.00], *p* < 0.0001)	(95% CI [65.1–95.6])	(95% CI [86.5–99.5])
Prediabetes: HbA1c ≥ 5.7*%* (13)	Group 1	0.98	96.9%	89.8%
		(95% CI [0.94–1.00], *p* < 0.0001)	(95% CI [92.4–99.2])	(95% CI [80.2–95.8])
	Group 2	0.99	100%	87.8%
		(95% CI [0.94–1.00], *p* < 0.0001)	(95% CI [92.0–100])	(95% CI [71.8–96.6])
Therapeutic goal: HbA1c < 7.0*%* (14)	Group 1	0.99	93.6%	100%
		(95% CI [0.95–1.00], *p* < 0.0001)	(95% CI [84.5–98.2])	(95% CI [86.3–100])
	Group 2	1.00	100%	100%
		(95% CI [0.86–1.00], *p* < 0.0001)	(95% CI [83.2–100])	(95% CI [54.1–100])
Therapeutic reinforcement: HbA1c ≥ 8.0*%* (15)	Group 1	0.99	94.4%	98.0%
		(95% CI [0.95–1.00], *p* < 0.0001)	(95% CI [81.3–99.3])	(95% CI [89.7–100])
	Group 2	1.00	100%	100%
		(95% CI [0.86–1.00], *p* < 0.0001)	(95% CI [76.8–100])	(95% CI [73.5–100])

Abbreviation: AUC, area under the ROC curve.

## 4. Discussion

In summary, our findings demonstrate strong agreement and linearity between HbA1c measurements obtained from alternative tubes (fluoride‐oxalate and lithium heparin) and standard K3‐EDTA tubes (*p* < 0.0001). No statistically significant overall mean bias was observed for either fluoride‐oxalate (−0.15%) or lithium heparin (−0.04%). Passing–Bablok regression confirmed the absence of systematic bias for lithium heparin; however, a slight systematic bias was detected for fluoride‐oxalate, as the 95% CI for the slope (0.99) and intercept (0.05) did not include 1 and 0, respectively. Additionally, regression analysis of the Bland–Altman plots revealed a proportional bias for both tube types, where differences varied positively with the mean. This indicates that the negative bias observed at lower concentrations tends to attenuate as HbA1c levels increase. Nevertheless, this bias is clinically negligible; as the log‐transformed measurement ratios (expressed via log‐transformed data on the *y*‐axis) remained tightly confined between 0.99 and 1.01 for both anticoagulants, confirming that these minor statistical deviations do not compromise clinical decision‐making.

Furthermore, it is widely accepted that a clinically significant change in glycemic control requires an absolute difference of at least 0.5% (in HbA1c units) between successive patient samples (16). This clinical requirement is fully satisfied in our study: The maximal relative deviation represented by the 95% LoA (which did not exceed ±2.77%) translates to an absolute HbA1c variation well below this threshold across standard clinical levels. Specifically, this relative limit corresponds to an absolute variation of only ±0.16% at the prediabetes threshold (5.7%), ±0.18% at the diagnostic threshold (6.5%), ±0.19% at the standard therapeutic target (7.0%), and ±0.22% at the poor glycemic control threshold (8.0%).

Regarding the identified outliers, the overall analytical agreement remained high, and these rare variations do not detract from the observed concordance between sample types. Indeed, when translating the maximum relative outliers (up to 7.9% for fluoride‐oxalate and 6.4% for lithium heparin) into absolute values, the discrepancies remain clinically minor. Across critical decision thresholds, the absolute differences stay within or extremely close to the 0.5% HbA1c relevance threshold (ranging from 0.35% to 0.55%). Consequently, these isolated variations are unlikely to affect routine clinical interpretation.

Moreover, HbA1c diagnostic performance at critical clinical thresholds remained robust for both alternative tubes compared to K3‐EDTA (*p* < 0.0001), effectively mitigating the risk of patient misclassification.

Given the absence of meaningful clinical differences, Vacutest fluoride‐oxalate and lithium heparin tubes provide reliable alternatives to standard K3‐EDTA tubes for HbA1c measurement on the Roche COBAS immunoassay platform. Consequently, due to potential methodology‐specific interferences, extrapolation to other methods (e.g., ion‐exchange chromatography or capillary electrophoresis) cannot be assumed reliable without independent validation.

This comparability of HbA1c measurements across different anticoagulants in whole blood collection tubes has been confirmed in the literature using various measurement methods. Within the scope of our research, we identified three studies that validate the use of anticoagulants other than EDTA for HbA1c measurement (5, 6, 17). Even for EDTA itself, Vrtaric et al. (18) documented the comparability of HbA1c results (*n* = 45) using Vacuette K2‐EDTA and K3‐EDTA tubes (Greiner Bio‐One, Austria) through a chemiluminescent microparticle immunoassay (CMIA) (ARCHITECT i2000SR, Abbott Laboratories).

Mailankot et al. (5) investigated the comparability of various anticoagulants (EDTA, sodium citrate, heparin, and fluoride) (Agappe Diagnostics) for HbA1c measurement using cation‐exchange high‐performance liquid chromatography (HPLC) (BioRad D10). No statistically significant differences were observed; however, the study population was too limited to assess the relevance of the results (six patients, including two diabetics). In a larger sample (*n* = 35), Boonlert et al. (6) compared HbA1c results using various anticoagulants (EDTA, lithium heparin, lithium heparin with glyceraldehyde, and sodium fluoride). Similarly, no statistically significant differences were observed among the tube types across both methods TINIA and HPLC.

Furthermore, Chakraborty et al. (17) published a study on the comparability of various anticoagulants (Vacutainer potassium EDTA tubes, sodium fluoride/sodium EDTA tubes, and Venosafe sodium fluoride/citrate‐buffered EDTA tubes) for HbA1c measurement (*n* = 104) using cation‐exchange HPLC (BioRad D10). Data analysis revealed statistically significant differences between the results attributable to the different types of anticoagulants. However, these differences were found to be smaller than the minimal clinically significant difference and were therefore deemed unlikely to have clinical implications.

Current recommendations for analytical performance targets for HbA1c measurement require an intralaboratory imprecision below 1.5% and an interlaboratory imprecision below 2.5%, ideally with no measurable bias (12). Our results strictly align with these standards, enhancing the relevance of our study to support a decision.

From a practical perspective, validating fluoride‐oxalate and lithium heparin tubes as reliable alternatives to K3‐EDTA may enhance routine laboratory workflow and improve resource management under real‐world local conditions. Specifically, it allows the laboratory to safely resolve common preanalytical nonconformities such as missing, hemolyzed, or broken K3‐EDTA tubes by using available alternative anticoagulants (fluoride‐oxalate or lithium heparin). This strategy minimizes blood collection volumes and unnecessary venipunctures, particularly in vulnerable patients, while yielding significant cost savings.

Our study has certain limitations that warrant acknowledgment. First, the presence of hemoglobin variants was not screened for and may influence HbA1c measurement results. In fact, the immunoinhibition used in the TINIA method eliminates potential interferences from the most common variants (Hb‐S and Hb‐C), although significant amounts of Hb‐F (> 10%) have been reported to cause an underestimation of HbA1c results (11, 19). Second, sample stability over time was not investigated, as this work did not evaluate the impact of prolonged storage or transportation delays on HbA1c measurements. Third, hemolysis indices were not assessed; although our routine workflow filters out visually unacceptable samples, undetected hemolysis represents a potential confounding factor that can interfere with immunoassay methods. Nevertheless, because our study was designed to evaluate paired samples from the same patient using an identical analytical method, any confounding factors such as hemoglobin variants, denatured fractions, or baseline hemolysis would have similarly affected both tubes, thereby preventing any systematic bias on the analytical difference attributable solely to the anticoagulant type.

Although EDTA is widely used as the standard anticoagulant for HbA1c measurement, its use is mainly based on historical laboratory practice rather than proven analytical superiority (20). This preference has remained deeply established because current diagnostic thresholds for diabetes were originally defined using the EDTA matrix. Given the robust comparability of alternative anticoagulants, this conventional practice warrants reconsideration.

We anticipate that these findings will assist other laboratories utilizing the same analytical platform in optimizing tube selection, thereby reducing costs without compromising quality. Ultimately, this work exemplifies the analytical prudence required to secure routine clinical workflows and establishes a practical reference framework for consulting similar platform‐specific studies during future technical transitions.

## 5. Conclusions

In conclusion, fluoride‐oxalate and lithium heparin tubes provide reliable alternatives to standard K3‐EDTA for HbA1c measurement on the COBAS INTEGRA 400 plus, as the detected proportional bias remains below the threshold of clinical relevance.

Within a comprehensive quality and cost‐effectiveness framework, the systematic requirement for EDTA tubes for HbA1c measurement appears unnecessary, as adopting these alternatives optimizes laboratory workflows and reduces patient blood collection.

However, due to potential methodology‐specific interferences, this reliability cannot be extrapolated to other analytical platforms without independent validation.

**Nomenclature**
HbA1c: glycosylated hemoglobin type A1c
BRISQ: Biospecimen Reporting for Improved Study Quality
EDTA: ethylenediaminetetraacetic acid
K3‐EDTA: tripotassium‐EDTA
ROC analysis: receiver operating characteristic analysis
CV: coefficient of variation
Hb: hemoglobin
CI: confidence interval

## Funding

No funding was received for this manuscript.

## Conflicts of Interest

The authors declare no conflicts of interest.

## Supporting information


**Supporting Information** Additional supporting information can be found online in the Supporting Information section. Supporting Information.

## Data Availability

Data are available upon reasonable request from the corresponding author.
