# Supplementary material for: Fluoride‐Oxalate and Lithium Heparin Anticoagulant Tubes Are Reliable Alternatives to EDTA for HbA1c Measurement
Source: Biomed Res Int. 2026 Aug 20;2026:7743935. doi: 10.1155/bmri/7743935 (PMC13491386; doi:10.1155/bmri/7743935)
Supplement: Supplementary file 1 [file BMRI-2026-7743935-s001.pdf]

Biological and Biomedical Sciences Data Reporting Checklist

At Wiley, we wish to improve the reproducibility and data quality of all our published content according to the FAIR principles. Please fill our this form as part of the submission process for all original research articles reporting investigations within the fields of biological and biomedical sciences.

For further information, please see Wiley's data sharing policy.

1. Statements and Declarations

In the following table, please mark the declarations made.

Have declarations been made in full as detailed in our guide to authors?	Please fill in all that apply	
Author contributions	A statement has been provided about the responsibility in the manuscript that specifies the contribution of every author. This is required for all manuscript types.	✔
Funding statement	Authors have stated how the research and publication of their article was funded, even if it was not funded externally and was performed as part of their employment.	✔
Conflict of interest	Authors have declared all relevant interests that could be perceived as conflicting.	✔
Cover letter	It is advisable (though not mandatory) to provide a cover letter detailing why the manuscript is appropriate for the journal, the overall importance of the work and the affiliation and contact information for the corresponding author.	✔

2. Materials, Systems, and Methods

In the following table, please select all of the materials/methods that are relevant to your work in the left column (by checking the box). Then, for each of these checked boxes in the left column, please provide all the requested information in the right column.

What materials, methods, systems are used?	Please fill in all that apply	
☐ Therapeutic agents, Drugs, drug-like compounds, probes, inhibitors	Toxicity analysis is provided	☐
	Selectivity analysis is provided	☐
	Formulation, including concentration and vehicle are provided	☐
☐ DNA, RNA, or peptide sequences	Sequences, accession or catalogue numbers of all DNA and RNA molecules, including plasmids, used are provided..	☐
☐ Proteins	Sufficient information to identify the proteins studied (e.g. protein name, organism of origin, accession number, and any modifications) are provided	☐
☐ Antibodies	Supplier name, lot number, clone name, and catalogue number for commercially available antibodies are provided	☐

☐ **Prokaryotes and fungi**	Source, including supplier name and catalogue number for commercially available prokaryotes and fungi are provided	☐
	Genus, species, strain, or RRID are provided	☐
☐ **Eukaryotic cell lines and primary cell cultures**	The cell line source (e.g. supplier name, catalogue number) are provided	☐
☐ **Animals or animal-derived materials**	An ethics statement that includes the name of the authority and the approval or accreditation number of the laboratory, project, or investigator is provided	This study complies with the PHS Policy on Humane Care and Use of Laboratory Animals ☐
	This study complies with the ARRIVE guidelines	The accession number or supplier name (including commercially available or biobank samples), catalogue number, clone number, or RRID are provided ☐
☐ **Human research participants**	An ethics statement that includes the name of the authority and the approval or accreditation number, of the laboratory, project, or investigator is provided	☐
☐ **Clinical trials**	Name of the authority that granted this exemption is provided	☐
	This study complies with the WMA Declaration of Helsinki – Ethical Principles for Medical Research Involving Human Subjects	☐
✔ **Human-derived materials**	Phase 2 and 3 clinical trials are reported in accordance with the CONSORT Guidelines	☐
**including blood, urine, feces, and serum;**	Tumor-marker studies are reported in accordance with the REMARK guidelines	☐
**testing of sensor or wearable technologies**	Human biospecimens studies are reported in accordance with the BRISQ guidelines	✔
**Human embryos and/or stem cells**	This study complies with the NIH Policy for Research involving Human Embryos	☐
	This manuscript maintains the anonymity of the study participants	✔
	Informed, written consent was obtained from all participants or their next of kin	☐

3. Experimental Design, Data Analysis, and Presentation

In the following table, please select all that apply to your study

☐ **Images of gels and/or blots**	Lanes have not been spliced together	☐
	Lanes from the same gel or blot have been spliced together, and the splicing is indicated in the figure legend and by a line on the figure	☐
	The corresponding molecular weights are indicated on gels and blots, wherever possible	☐
	Loading controls were run on the same gel	☐
☐ **Flow Cytometry**	Gating strategy has been shown	☐
	Laser wavelengths are clear and readable.	☐
☐ **PCR and qRT-PCR**	Primers used have been listed	☐
	Genes used for qRT-PCR have been listed	☐
☐ **Fluorescent microscopy**	Fluorescent probes/dyes/protein used are labeled on the figure itself above/adjacent to the corresponding images.	☐
	Scale bar is shown and readable.	☐
✔ **Figures** (general)	All Figures are presented with the highest clarity and resolution	✔
	Unprocessed images will be made available upon request	☐
✔ **Charts/graphs**	Individual data points are shown (whenever possible).	✔
	Statistical significance is shown.	✔
	Error bars are shown.	✔
✔ **Experimental design: e.g. Randomization, Blinding, Data Exclusion**	Criteria upon which the samples/organisms/participants were allocated into experimental groups are provided	✔
	If this allocation was random, the measures used to control covariates are provided	☐
	Investigators were blinded to the group allocation during data collection and/or Analysis	☐
	Blinding was not possible or not relevant	✔

✔ **Statistics**	Sample size (n) for each experimental group in each experiment is provided	✔
	Number and type (i.e. biological or technical) of replicates performed in each experiment and relevant information on the successful attempts at replication is provided	✔
	The name(s) of the statistical test(s) used	✔
	The name and version of the software used to analyze significance	✔
	Significance is defined for each experiment	✔
✔ **Figure legends**	The number and type of repeated experiments and sample size within is provided	✔
	Definition of error bars (i.e. confidence interval, standard deviation, or standard error or the mean), statistical test used is provided	✔
	Scale bar size and magnification are clearly shown	✔

This Biological and Biomedical SciencesData Reporting Checklist is made available under the Creative Commons Attribution (CC-BY) License, which permits use, distribution and reproduction in any medium, provided the original checklist is properly cited. Please consult the related article for the license terms applicable to the article.
